# Isolation and Characterization of Rabbit Spermatogonial Stem Cells as a Promising Animal Genetic Resource for Biodiversity Protection

**DOI:** 10.3390/biom16091278

**Published:** 2026-09-03

**Authors:** Jaromír Vašíček, Andrej Baláži, Andrea Svoradová, Jakub Vozaf, Miroslav Bauer, Marián Tomka, Lucia Olexiková, Peter Chrenek

**Affiliations:** 1 Division of Animal Genetics and Reproduction, Department of Animal Production, National Agricultural and Food Centre, Hlohovecká 2, 951 41 Lužianky, Slovakia; andrej.balazi@nppc.sk (A.B.); jakub.vozaf@nppc.sk (J.V.); miroslav.bauer@nppc.sk (M.B.); lucia.olexikova@nppc.sk (L.O.); peter.chrenek@nppc.sk (P.C.); 2 Institute of Biotechnology, Faculty of Biotechnology and Food Science, Slovak University of Agriculture in Nitra, Tr. A. Hlinku 2, 949 76 Nitra, Slovakia; marian.tomka@uniag.sk; 3 Division of Animal Nutrition and Small Livestock, Department of Animal Production, National Agricultural and Food Centre, Hlohovecká 2, 951 41 Lužianky, Slovakia; andrea.svoradova@nppc.sk

**Keywords:** rabbit, spermatogonial stem cells, cell culture, GDNF, GFRα-1, FGF2, molecular profiling, TEM

## Abstract

The continual spermatogenesis throughout adulthood is ensured by a rare and unique cell group named spermatogonial stem cells (SSCs), which undergo self-renewal and/or differentiate into sperm cells. SSCs also become a promising genetic source for the protection of animal biodiversity. However, the isolation and culture of SSCs in vitro is still a big challenge and poorly explored in rabbits. The main objective of this study was to isolate, culture, and deeply characterize SSCs obtained from rabbit testes. Briefly, rabbit testicular tissue was mechanically and enzymatically dissociated, and obtained testicular somatic and germ cells were cultured for a short term in culture media supplemented with specific molecular factors maintaining SSC self-renewal and proliferation (GDNF, GFRα-1, FGF2, etc.). Immunofluorescent and PCR techniques were used for molecular profiling of cultured SSCs, while TEM analysis revealed their ultrastructure. After a few weeks, round and grape-like SSC colonies emerged, growing on the feeder cell layer. Rabbit SSCs showed positive staining for DBA, GFRA1, PLZF, RET, PGP9.5, DAZL, and DDX4. Increased expression of additional SSC markers was noticed using RT-qPCR and dd PCR (RET, PLZF, PGP9.5, DAZL, DDX4, CDH1, CD9, CD14, CD90, c-kit, ALDH, SSEA-4, SALL4, OCT4, and SOX2), while ultrastructure typical for primitive undifferentiated cells was observed under TEM. In conclusion, we successfully established a method for rabbit SSC isolation, culture, and phenotyping, which might facilitate their collection for further cryopreservation. However, the self-renewal, proliferative, and differentiation capacities of cultured SSCs still need to be confirmed through an in vivo SSC transplantation experiment.

## 1. Introduction

Spermatogonial stem cells (SSCs) are a unique cell population located within the testis tissue that sustains spermatogenesis and fertility of males throughout their lives by undergoing self-renewal and continuous production of mature sperm cells through complex processes of spermatogonia differentiation, through which their specific genetic information is transferred to the future generation [1]. Although first functional SSCs can already be found in mice 3 to 8 days or in humans 3 to 12 months after birth [2], they are very rarely present in adult testes of animals (only 0.01–0.03% of testicular cells) [3]. Despite their rare presence, SSCs have great therapeutic potential and can be used for the genetic conservation of endangered species of wildlife animals or even valuable rare livestock breeds through their cryopreservation and/or further by the method of testis tissue xenografting [1].

However, the isolation and culture of SSCs in vitro is still a big challenge, since several approaches could be used with different rates of success to obtain a pure SSC population. Moreover, the in vitro culture conditions must mimic the testis tissue niche. The most often studied enrichment of SSCs is based on fluorescence-activated cell sorting (FACS) or magnetic-activated cell sorting (MACS), separation of SSCs using the linear gradient of bovine serum albumin (Staput velocity sedimentation) or via the selection of SSCs using different components of extracellular matrix (differential plating) [4]. In fact, the identification and isolation of SSCs using standard sorting techniques (FACS or MACS) based on a universal molecular marker is complicated and not uniform for different animal species. Usually, the phenotype of SSCs originating from different animals is species-specific, and thus a combination of several markers is used to identify a pure SSC population. Therefore, another commonly used method of SSC purification is based on the specific affinity of testicular cells to different extracellular matrix components, such as somatic cells (Sertoli cells, Leydig cells, etc.) to gelatine and lectin, or SSCs that can bind to laminin via α- and β-integrin receptors [4,5]. This method has been used to enrich up to 75% of SSCs within the testicular cells obtained from bull, ram, or boar [6].

According to the literature, different culture conditions are used to successfully proliferate animal SSCs by maintaining their self-renewal ability for short or even long term. Basically, the most important prerequisite of SSC proliferation is the type of culture medium, mainly DMEM or StemPro medium [7,8] and its components such as serum [9] and essential growth factors. However, serum contains undefined factors that might negatively affect the survival of SSCs in culture [10]. Therefore, a serum-free culture system has been established for these stem cells originating from either rodents [10,11,12,13], or non-rodents [9,14,15] for the better maintenance of SSC culture. After the discovery that a paracrine factor, GDNF (glial cell line-derived neurotrophic factor), secreted by Sertoli cells located in the SSC niche, promotes their self-renewal and maintenance in vivo [16], GDNF became a major molecular component in serum-free culture media for short-term and long-term in vitro SSC proliferation [10,17,18,19,20]. GNDF signaling acts on the undifferentiated type of spermatogonia via a transmembrane tyrosine kinase RET after binding of GDNF to its specific cell surface molecular receptor GFRα-1, resulting in a GDNF/GFRα-1/RET complex, which promotes self-renewal of SSCs [2,21]. Fibroblast growth factor 2 (FGF2) is also secreted by Sertoli cells and, according to different studies, plays a minor supportive role in SSC self-renewal, as the addition of this extrinsic factor simultaneously with GDNF supports proliferation of SSCs in rodents such as mice, rats, or hamsters [10,12,22,23]. The last prerequisite of successful SSC culture is a suitable feeder cell layer. Although feeder-free culture systems for SSCs have also been developed [9,11,24], different feeder cells are used for short-term or long-term proliferation of SSCs, such as mitomycin C-treated STO and C166 cells, somatic testicular cells, or Sertoli cells themselves [21,25,26].

On the other hand, to replicate the suitable conditions for in vitro SSC culture or to identify pure SSCs obtained from different animal species by one specific molecular marker may be quite complicated according to different SSC phenotypes found in the literature [27]. Thus, further research focused on animal SSCs is still required and strongly desirable to obtain novel valuable information. At the present, a combination of molecular markers, such as PLZF (promyelocytic leukemia zinc finger), PGP9.5 (protein gene product 9.5), CD90/Thy-1 (cluster of differentiation 90/thymocyte differentiation antigen 1), GFRα1 (GDNF family receptor alpha-1), DBA (Dolichos biflorus agglutinin), VASA/DDX4 (DEAD-box polypeptide 4), NANOG (transcription factor related to the pluripotency of stem cells), POU5F1/OCT4 (POU domain, class 5), DAZL (deleted in azoospermia-like protein), ITGA6/CD49f (integrin subunit alpha 6/cluster of differentiation 49f), and CDH1 (cadherin 1), are used to identify and/or isolate SSCs from several farm animals, mainly horses, cattle, sheep, goats, and pigs [4]. The expression pattern of these markers differs among the species; thus, the search for novel phenotype-specific markers identifying the SSCs of farm animals is of great importance. Recently, we have adopted an aldehyde dehydrogenase (ALDH) as a universal “stemness” marker for several types of rabbit stem and progenitor cells [28,29] as well as for chicken primordial germ cells [30] obtained from Slovak native farm animal breeds, which have been later cryopreserved as valuable genetic resources in our stem cell bank. The expression of this promising stem cell marker by SSCs has been studied only in mice [31] or dunnarts [32]. Unfortunately, as far as we know, only a few studies have focused on rabbit SSCs [21,33,34]. In one of them, a long-term culture of rabbit SSCs was established using a C166 feeder layer and addition of GDNF and FGF2 factors. The authors also demonstrated the expression of several above-mentioned SSC markers [21]. However, a specific isolation method of pure rabbit SSCs, or a culture system based on different feeder layers as well as deeper characterization of rabbit SSC phenotype and their ultrastructure has been poorly explored.

Thus, the aim of this study was to isolate rabbit SSCs by cultivating them on an adherent somatic testicular cell feeder layer in a specific culture medium supplemented with molecular factors essential for SSC maintenance and self-renewal and to comprehensively characterize rabbit SSC phenotype at the molecular level using different PCR techniques in order to find specific molecular markers for their future identification or direct isolation.

## 2. Materials and Methods

### 2.1. Animals and Testicular Cell Isolation

Healthy rabbit males of the New Zealand White (NZW) line (*n* = 7) at the age of 3 months were used for the experiments. Animals were reared at NPPC—Department of Animal Production as described previously [35]. All experimental work was performed with approval of the Ministry of Agriculture and Rural Development of the Slovak Republic no. SK U 18021 (22 December 2021) in accordance with the ethical guidelines presented in Slovak Animal Protection Regulation (RD 377/12), which conforms to the Code of Ethics of the EU Directive 2010/63/EU for animal experiments. No other approval was required for this study, since the biological material (testes) used in the experiments was considered biological waste from rabbit meat production. The work has been reported in line with the ARRIVE guidelines 2.0.

Testis tissue samples were obtained from humanely sacrificed rabbits. Testicular cells (TCs) containing somatic and germ cells were isolated from the tissue by the combination of mechanical and enzymatic dissociation. Briefly, testes were decapsulated by careful removal of *tunica albuginea*, testis tissue was cut into small fragments using scissors, and the fragments were washed for 5 min. at room temperature at 300× *g* with a PBS (without Ca and Mg; Serana, Pessin, Germany) containing 5% antibiotics (penicillin/streptomycin/amphotericin B mixture; PAN-Biotech, Aidenbach, Germany). Then, tissue fragments were enzymatically digested with 0.2% collagenase type I (Merck, Darmstadt, Germany) for 1 h at 37 °C with constant shaking. To neutralize the enzymatic solution, low-glucose DMEM medium (Thermo Fisher Scientific, Waltham, MA, USA) supplemented with 10% fetal bovine serum (FBS; Merck) was added to the tube, and the cell suspension was immediately filtered through a 100 µm cell strainer to remove the undigested tissue, followed by filtration through a 40 µm cell strainer to obtain a single-cell population. TCs were centrifuged at 300× *g* for 5 min, then the cell pellet was resuspended in hemolytic solution to lyse any remaining red blood cells, and cells were centrifuged again.

### 2.2. In Vitro Culture of Rabbit SSCs

Afterward, TCs were diluted in high-glucose DMEM culture medium (Thermo Fisher Scientific) containing 1% antibiotics (PAN-Biotech) and other specific growth factors described below, seeded into 60 mm Petri dish or 6-well culture plate at a density of 2.5 × 10^5^ cells/cm^2^, and cultured for one week at 37 °C and 5% CO_2_ to establish SSC colonies growing on a monolayer formed by somatic testicular cells. The specific culture medium was prepared according to Valdivia et al. [7] and Kubota et al. [21] with some modifications, supplemented with 1% FBS (Merck), 0.29 mg/mL of L-glutamine, 10 µL/mL nonessential aminoacid MEM solution, 1 µL/mL insulin–sodium selenite–transferrin, 1 µL/mL lactic acid (all from Merck), 1 mM pyruvic acid (Serana), 0.5% bovine serum albumin (PAN-Biotech), 10 ng/mL recombinant human GDNF, 1 ng/mL recombinant human FGF2 (both from Thermo Fisher Scientific), and 100 ng/mL recombinant rat GFRα-1 (R&D Systems, Minneapolis, MN, USA). Cell morphology was monitored during the culture under a Zeiss Axio Observer.Z1/7 microscope (Carl Zeiss Slovakia, Bratislava, Slovakia). After one week of culture, when adherent cells reached more than 90% confluency and SSC colonies emerged in cell culture, cells were harvested using 0.05% Trypsin-EDTA (Thermo Fisher Scientific). Cell count and viability were assessed using an EVE^TM^ Automatic cell counter (Nano Entek, Seoul, Republic of Korea) by trypan blue staining. After washing, cells were diluted in culture medium and reseeded as passage 1 at a density of 1.3 × 10^5^ cells/cm^2^ for another week of culture. After that time, some SSC colonies were collected by gently washing the cell culture and dissociated using 0.25% Trypsin-EDTA (Thermo Fisher Scientific) to get a single-cell population for further analyses (cell viability, PCR, TEM, and other analyses). For flow-cytometric analysis of ALDH expression, SSC colonies together with somatic testicular cells at passage 1 were used. Since SSC colonies exhibited variable proliferation rates with different yields, we co-cultured some samples till passages 2 and 3 to get enough colonies for immunofluorescence analysis by confocal microscopy.

### 2.3. Molecular Profiling of Rabbit SSCs Using PCR Analyses

At least one million cultured rabbit adherent somatic testicular cells and SSCs (passage 1) were used to isolate RNA and prepare cDNA for subsequent PCR analyses as described previously [29]. To analyze expressions of surface and intracellular markers, we used primers summarized in Table 1, which have been published previously or designed de novo using the Primer-BLAST at the NCBI website [36].

Expression of various potential SSC surface and intracellular markers (CD9, CD14, CD29, CD49f, CD90, c-kit, CDH1, SSEA-4, ALDH, GFRα1, RET, PLZF, PGP9.5, DDX4, DAZL, SALL4, NANOG, OCT4, and SOX2) and Sertoli cell markers (Vimentin and SOX9) at the mRNA level in cultured rabbit samples was monitored using a quantitative real-time PCR (qPCR). The PCR reaction was performed as described in our previous study [28]. Beta-2-microglobulin (B2M) was used as a housekeeping gene to normalize the relative expression of observed markers. B2M served as a reliable internal reference control to normalize qPCR gene expression data in different SSC culture analyses [41,42]. The expression level of analyzed markers in adherent somatic cells was considered as 1 to better indicate the marker expression changes in cultured rabbit SSCs. PCR products of selected markers (GFRα1, RET, PLZF, PGP9.5, CDH1, DDX4, DAZL, SALL4, ALDH, NANOG, OCT4, and SOX2) were electrophoretically separated on a 2% agarose gel in TAE buffer to check the specific band size.

For absolute quantification of selected SSC marker expression (GFRα1, RET, PLZF, PGP9.5, CDH1, DDX4, DAZL, SALL4, ALDH, NANOG, OCT4, and SOX2), we performed digital droplet PCR (ddPCR) analysis as described previously [28] using a QX200 Droplet Digital PCR System (Bio-Rad, Hercules, CA, USA). The resulting marker expression was displayed as a percentage ratio of positive droplet count to the total count of droplets in the sample.

### 2.4. Flow-Cytometric Analysis of ALDH Expression

At least 10^6^ rabbit SSCs co-cultured with somatic testicular cells (passage 1) were analyzed by flow cytometry for the specific expression of aldehyde dehydrogenase using the ALDEFLUOR^TM^ kit (STEMCELL Technologies, Vancouver, BC, Canada) as described previously [30]. To analyze stained samples, a CytoFLEX S flow cytometer (Beckman Coulter, Indianapolis, IN, USA) equipped with CytExpert 2.6 software (Beckman Coulter, Inc., 2011–2023) was used to acquire at least 10,000 cells per sample in the FITC channel (525/40). Obtained data were analyzed using FlowJo™ Software v10.10.0 (BD Biosciences, San Jose, CA, USA).

### 2.5. Detection of SSC Markers Using Confocal Microscopy

Rabbit SSC samples from passage 1 or 2 were seeded on the round coverslips at the bottom of the Nunc™ 4-well dishes (Thermo Fisher Scientific). After forming colonies, cells were gently washed twice with PBS containing Ca and Mg. Then, colonies were fixed, permeabilized, and stained overnight with specific antibodies and reagents as described in our previous study [28]. To detect expression of specific SSC markers, FITC-conjugated DBA lectin (at final concentration of 100 µg/mL; Thermo Fisher Scientific) and primary mouse monoclonal antibodies against GFRα1 (clone 81401, 1:50; R&D Systems), RET (clone 132507, 1:100; R&D Systems), PLZF (clone D-9, 1:50; Santa Cruz Biotechnology, Santa Cruz, CA, USA), PGP9.5 (clone 13C4, 1:100; Origene Technologies, Rockville, MD, USA) and rabbit polyclonal antibodies against DDX4 (1:50, orb156544; Biorbyt, Cambridge, UK), and DAZL (1:100, ab13840; Abcam, Cambridge, UK) were used. After washing on the following day, cells were incubated with polyclonal goat anti-mouse (STAR117F; Bio-Rad) and anti-rabbit (Bio-Rad) FITC-conjugated secondary antibodies and then mounted on the microscope slides as described previously [28]. Samples were analyzed under the laser scanning confocal microscope LSM 700 (Carl Zeiss Slovakia).

### 2.6. Ultrastructural Analysis of Rabbit SSCs Using Transmission Electron Microscopy

Cultured rabbit SSCs (passage 1) were processed for transmission electron microscopy (TEM) according to the methodology of Duranova et al. [43]. Ultra-thin sections were collected on the grids, contrasted with uranyl acetate and lead citrate, and examined under a JEOL JEM-1400 transmission electron microscope (JEOL, Tokyo, Japan) at the Laboratory of Electron Microscopy at the Biology Centre CAS (České Budějovice, Czech Republic).

### 2.7. Statistical Analysis

Data obtained from analyses were evaluated using GraphPad Prism version 9.5.1 for Windows (GraphPad Software, San Diego, CA, USA) with one-way ANOVA (Fisher’s LSD test) and descriptive statistics. Results are expressed as the mean ± SD. *p*-values at *p* < 0.05 were considered statistically significant.

## 3. Results

### 3.1. Cell Morphology and Viability

**Figure 1:**
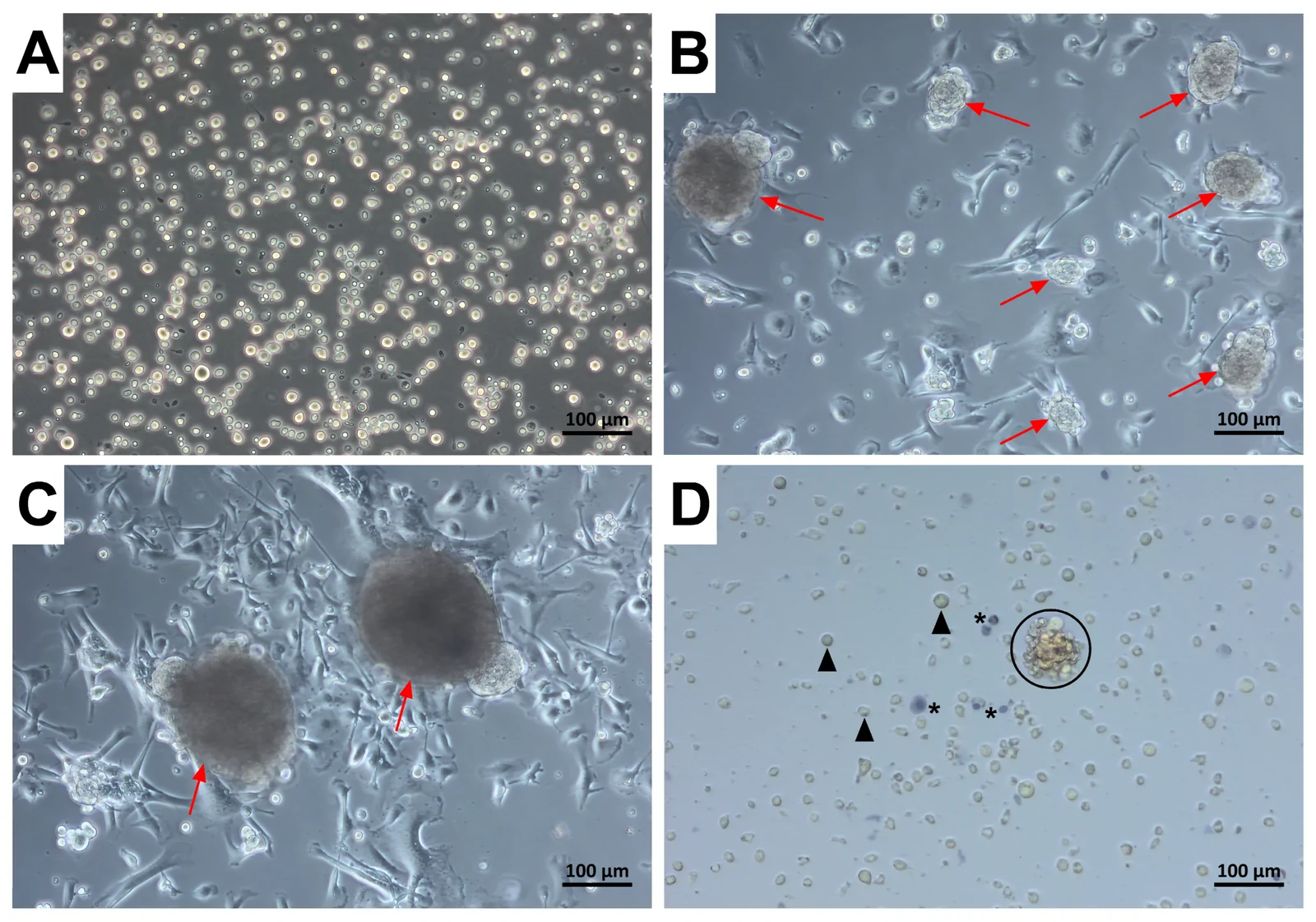
Morphology and viability of cultured rabbit testicular cells. Cells exhibited typical round morphology after seeding into culture vessels (**A**). Germ cells at passage 1 formed typical round and grape-like colonies of different sizes (red arrows) growing on the adherent somatic cells (**B**,**C**). Viability of round rabbit germ cells of different sizes (black arrowheads) after dissociation of rabbit SSC colonies from passage 1 with trypsin-EDTA, stained with trypan blue dye (dead cells stained with dye are marked with asterisks). Undissociated SSC colony bordered by a black circle (**D**) observed under a Zeiss Axio Observer.Z1/7 microscope (magnification at 100×; scale bar = 100 μm).

Immediately after seeding, the majority of round somatic testicular cells adhered to the bottom of culture vessels, showing spindle-shaped morphology, and started to proliferate rapidly to form a monolayer at the end of the first week of culture. During this time, the rabbit SSC colonies of different sizes and typical round shapes emerged, growing on the surface of adherent cells (Figure 1). Moreover, rabbit SSCs maintained high viability over 90% during the whole culture from passage 1 to passage 3 (91.1 ± 5.5%, 90.7 ± 4.2% and 91.0 ± 3.6%, respectively).

### 3.2. Molecular Markers Expressed by Rabbit SSCs

**Figure 2:**
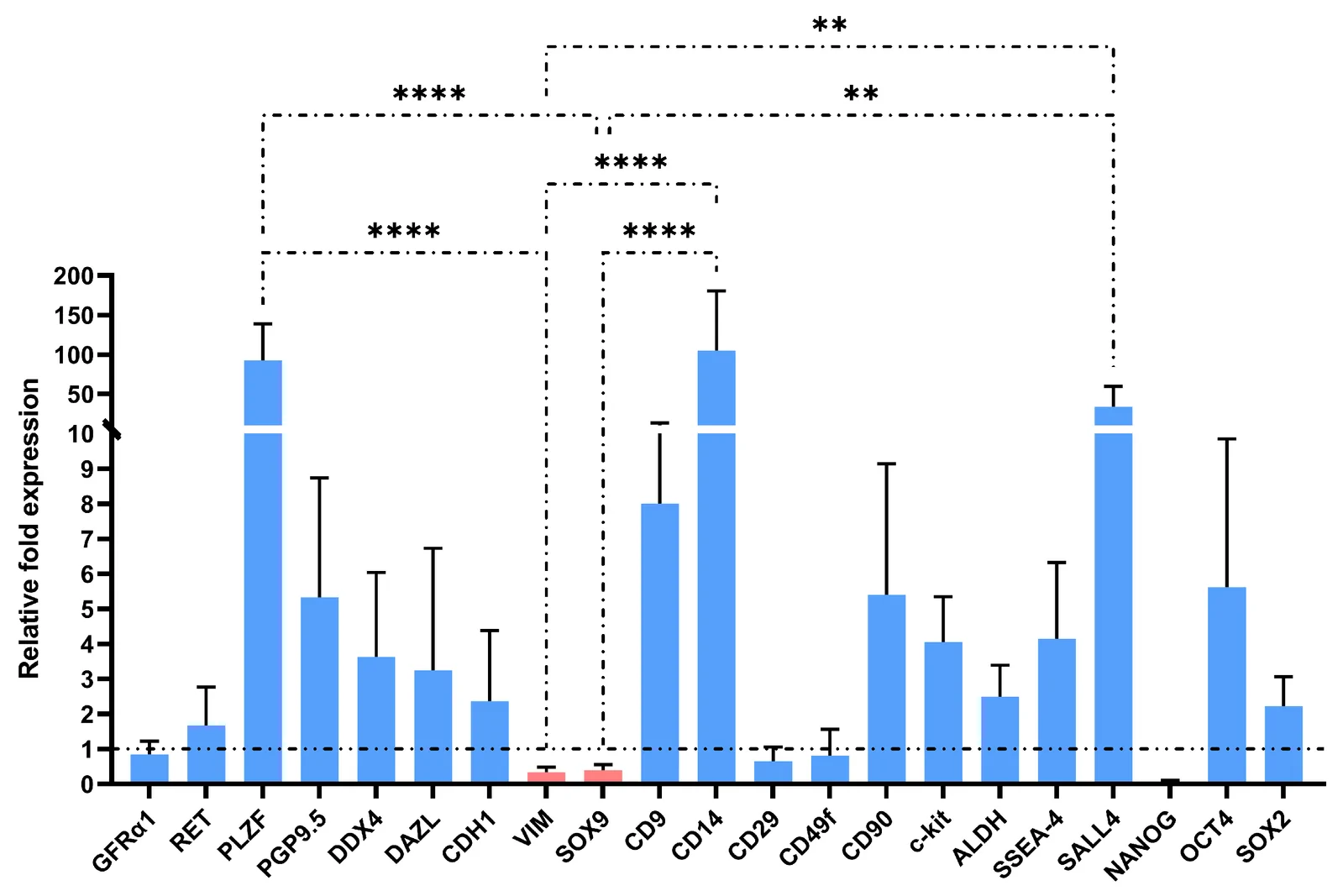
Relative expression of molecular markers at the mRNA level analyzed using qPCR in rabbit SCC samples (passage 1). The expression level of analyzed markers in adherent somatic testicular cells was considered as 1. The data from six biological replicates are expressed as the mean ± SD and compared to the relative expression of adherent Sertoli cell markers (Vimentin and SOX9); **—difference is statistically significant at *p* < 0.01; ****—difference is statistically significant at *p* < 0.0001.

We used PCR techniques to detect specific molecules at the mRNA level of rabbit SSCs (passage 1). According to qPCR, rabbit SSCs exhibited increased expression of several markers such as RET, PLZF, PGP9.5, DDX4, DAZL, CDH1, CD9, CD14, CD90, c-kit, ALDH, SSEA-4, SALL4, OCT4, and SOX2, while other markers like Sertoli cells markers (Vimentin and SOX9), CD29, CD49f, and NANOG were decreased, or not changed (GFRα1) when compared to their expression in adherent somatic cells. Moreover, highly significant differences were found for the expression of PLZF, CD14 (*p* < 0.0001), and SALL4 (*p* < 0.01) in comparison to the expression of Sertoli cell markers, Vimentin and SOX9 (Figure 2).

**Figure 3:**
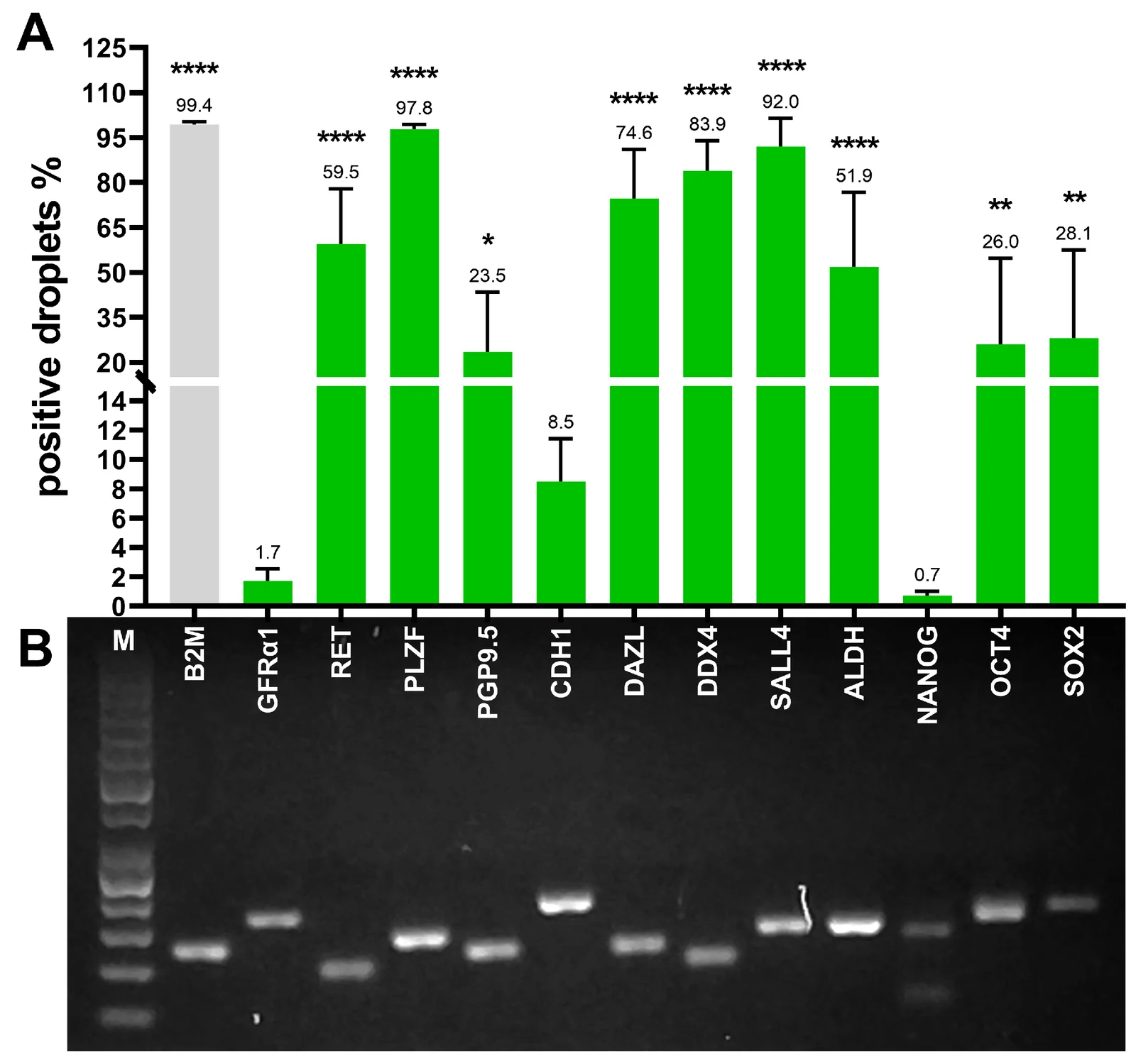
Expression of molecular markers at the mRNA level analyzed using gel electrophoresis of qPCR products and ddPCR in rabbit SCC samples (passage 1). The absolute expression of selected markers was quantified using ddPCR. The data from six biological replicates are expressed as the mean ± SD and compared to the lowest expressed molecular marker (NANOG); *—difference is statistically significant at *p* < 0.05; **—difference is statistically significant at *p* < 0.01; ****—difference is statistically significant at *p* < 0.0001 (**A**). Qualitative expression of selected molecular markers in agarose gel. Lane 1—50 bp DNA ladder (M); lane 2—B2M (118 bp); lane 3—GFRα1 (150 bp); lane 4—RET (93 bp); lane 5—PLZF (125 bp); lane 6—PGP9.5 (108 bp), lane 7—CDH1 (168 bp), lane 8—DAZL (108 bp), lane 9—DDX4 (93 bp), lane 10—SALL4 (133 bp), lane 11—ALDH (135 bp), lane 12—NANOG (122 bp), lane 13—OCT4 (149 bp), and lane 14—SOX2 (152 bp) (**B**).

By visualization of qPCR products of selected markers (GFRα1, RET, PLZF, PGP9.5, CDH1, DDX4, DAZL, SALL4, ALDH, NANOG, OCT4, and SOX2) using gel electrophoresis, bands of specific sizes were found, thus verifying their expression, although with different levels (Figure 3B). The same markers selected for ddPCR analysis displayed significantly higher expression of RET, PLZF (*p* < 0.0001), PGP9.5 (*p* < 0.05), DDX4, DAZL, SALL4, ALDH (*p* < 0.0001), OCT4, and SOX2 (*p* < 0.01) compared to the low-expressed NANOG (Figure 3A).

### 3.3. Expression of Aldehyde Dehydrogenase and Other Potential SSC Markers in Rabbit Testicular Somatic and Germ Cells

**Figure 4:**
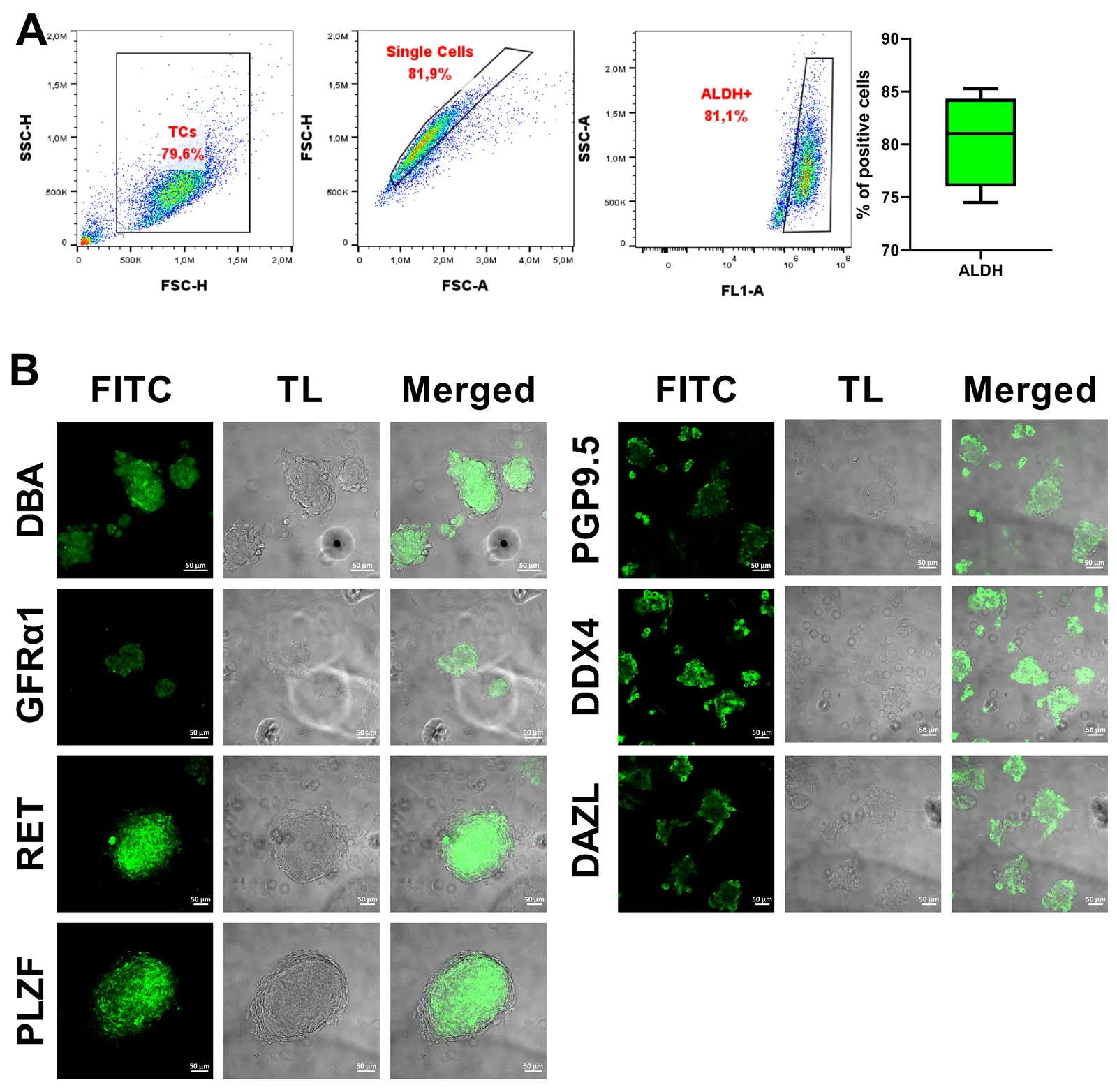
Immunofluorescent analysis of selected potential SSC markers expressed by rabbit SSC co-cultured with somatic testicular cells. Illustrative flow-cytometric dot plots show gating strategy and ALDH analysis using a specific ALDEFLUOR kit (TC—testicular cells). The data from four biological replicates are expressed as the mean ± SD (**A**). Rabbit SSC colonies in culture expressing several markers analyzed by laser scanning confocal microscope Zeiss LSM 700 (FITC—green channel, TL—transmitted light; magnification at 200×; scale bar = 50 μm) (**B**).

Flow cytometry revealed high expression (over 80%) of ALDH in rabbit somatic testicular cells and SSC co-culture, showing it to be strongly found in both adherent somatic and germ cells of rabbit testicular cells cultured in vitro (Figure 4A). Confocal fluorescent microscopy verified the expression of other positive markers by rabbit SSC colonies (passage 2 or 3) at the protein level using specific antibodies against GFRα1, RET, PLZF, PGP9.5, DDX4, and DAZL, as well as positivity to DBA staining (Figure 4B).

### 3.4. Ultrastructure of Cultured Rabbit SSCs

**Figure 5:**
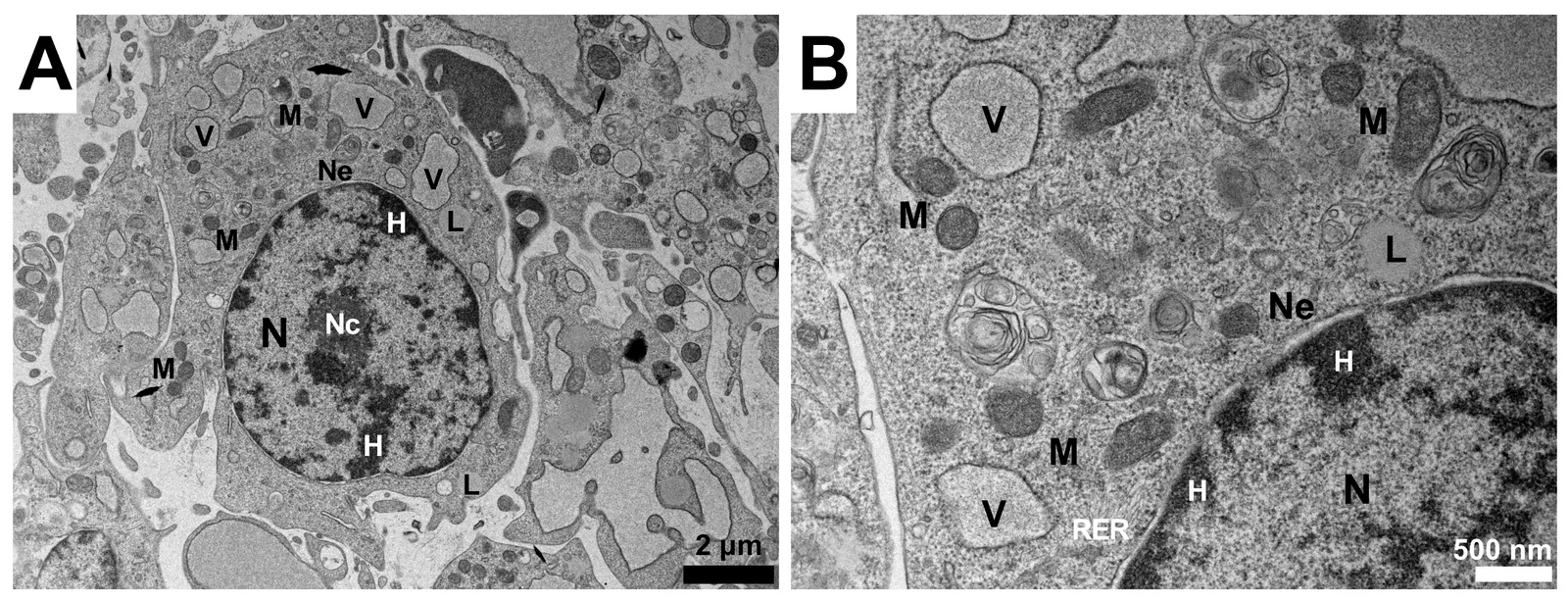
Electron micrographs of cultured rabbit SSCs. TEM micrographs of an entire cell (**A**) with an oval shape with large round nucleus (N) surrounded by double nuclear envelope (Ne) and containing small nucleolus (Nc) and dense heterochromatin (H), while many round mitochondria (M) and different vacuoles (V), and few lipid droplets (L) and rough endoplasmic reticulum (RER) are present throughout the cytoplasm (**B**) (JEM-1400, JEOL, Tokyo, Japan).

Observation under a transmission electron microscope revealed the typical morphology of spermatogonial stem cells, exhibiting a large round nucleus with small and dark heterochromatin areas on the margin and a small compact nucleolus in the center surrounded by mainly spherical mitochondria, vacuoles of different sizes, few lipid droplets, and rough endoplasmic reticulum in the cytoplasm (Figure 5).

## 4. Discussion

To our knowledge, this is the first study describing isolation and culture of rabbit SSCs using somatic testicular cells as a feeder layer, as well as detailed characterization of their phenotype and discovery of their ultrastructure, which might help to accelerate further studies utilizing rabbit spermatogonial stem cells. The culture system for the proliferation of rabbit testicular somatic and germ cells established here consists of high-glucose DMEM medium with a low serum level (only 1%) and supplemented with specific components described in the methods. A similar composition of culture media with some differences was used to establish SSC culture in alpacas [7,44,45], mice [26], sheep [46], humans [8], or pigs [25]. Nevertheless, the most important molecular factors found in all the published studies are GDNF, FGF2, and GFRα-1 supplements. According to Kubota et al. [21], rabbit testicular germ cells might be long-term cultured for 7 months with 40 ng/mL GDNF, 300 ng/mL GFRα-1, and 1 ng/mL FGF2, while maintaining their constant proliferation even when GDNF and GFRα-1 concentrations are halved. We observed the formation of typical round and grape-like SSC colonies or clusters in a short-term culture system under lower concentrations of GDNF and GFRα-1 (10 and 100 ng/mL, respectively), which exhibited constant proliferation and high viability during the whole culture period. The same GDNF concentration has been used for the expansion of human [8] or porcine [25] SSCs in co-culture with somatic testicular cells. Thus, we might assume that the somatic testicular cells (Sertoli cells, etc.) in co-culture with SSCs produce intrinsic GDNF, which in combination with extrinsic supplementation of GDNF allows expansion of rabbit SSCs. However, this hypothesis cannot be verified without further experiments and measurement of the level of GDNF produced by feeder cells, which was not assessed in this study. In fact, it is known that SSC self-renewal is regulated by the GDNF/GFRα-1/RET complex [2,10,21,47]. The increased level of GDNF resulted in the accumulation of undifferentiated spermatogonia. On the other hand, the loss of GFRα-1/RET receptors dramatically decreases the presence of SSCs. Basically, undifferentiated spermatogonia exhibit high levels of GFRα-1, which is continuously decreased by further differentiation [47]. Expression of GFRα-1 in undifferentiated spermatogonia is regulated by GDNF itself [48].

In this study, we observed expression of GFRα-1 on the cell surface of cultured rabbit SSCs using a specific antibody, which has been successfully used to identify rabbit spermatogonia in a previous study using flow cytometry [21]. On the contrary, GFRα-1 expression at the mRNA level of our rabbit SSC samples was not elevated and was comparable to somatic testicular cell expression. According to the literature, the external addition of soluble GFRα1 may influence marker expression and endogenous receptors in SSCs, thus promoting down-regulation or shifting cells toward differentiation pathways. Although soluble GFRα1 can bind extracellular GDNF, allowing it to be available for RET receptor and to maintain SSC self-renewal, changes in the stoichiometric balance of GDNF/GFRα1/RET complex may drive SSCs out of their self-renewal state, leading to a secondary reduction in endogenous stem cell markers like GFRα1 [49,50,51]. On the other hand, the undifferentiated spermatogonial pool is not uniform, but functionally and molecularly diverse, containing a mixture of true stem cells and committed progenitors that express varying levels of markers like GFRα1 [52,53]. Anyway, an increased expression of this marker in pure SSCs should be expected at the mRNA level when compared to the somatic testicular cells. Therefore, according to our PCR results, we have carefully checked again the nucleotide sequence of the predicted rabbit GFRα1 transcript variant (XM_051824180.1) used for the design of specific primers. Unfortunately, we found a recent update in this sequence (XM_051824180.2) that resulted in only 95% identity with the forward primer. Thus, we assumed that this issue might decrease the efficiency of amplifying the GFRα1 transcript in the analyzed rabbit SSC samples.

On the other hand, RET and other typical SSC markers like PLZF, PGP9.5, DDX4, and DAZL have been abundantly expressed in rabbit SSC colonies, as proved by immunofluorescent staining or PCR analysis at the mRNA level. Similar expression was observed in rabbit germ cells by immunocytochemistry (GFRα1, RET, PLZF, DDX4, and OCT4) or by flow cytometry (CD9, CD49f, and CD90) [21]. According to the literature, mouse spermatogonia also displayed the expression of GFRα1, CD9, CD29, CD49, and CD90 [54]. Although we did not observe changes in the expression of CD29 or CD49f by rabbit SSCs in our study compared to somatic testicular cells (feeder layer cells), expression of CD9 and CD90 was elevated at different levels. Nevertheless, CD29 and CD49f integrins, as important adhesion molecules involved in SSC homing and interactions with their niche [44] may also be found in Sertoli cells [55,56], which might explain the non-elevated expression of these markers in rabbit SSCs. All above-mentioned molecular markers have been found with some differences in equine, bovine, ovine, caprine, or porcine SSCs, including DBA and NANOG [4,57,58,59]. While DBA, which specifically recognizes glycan epitopes on SSCs [44], was positively detected in our rabbit SSCs, these germ cells did not show NANOG expression. Nevertheless, it was reported that expression of NANOG is absent in cultured SSCs [60,61] whereas it can be rarely found in adult mouse testes [62] or neonatal pig or cattle testes in vivo together with OCT4 [63,64]. Here, we noticed that other pluripotency-related transcription factors, OCT4 and SOX2, were upregulated in cultured rabbit SSCs, thus pointing out their undifferentiated state.

Additionally, cultured rabbit SSCs exhibited upregulation of other promising molecular markers like CDH1, CD14, ALDH, c-kit, SSEA-4, and SALL4. CDH1 expression has also been reported in mouse and rat undifferentiated spermatogonia [65,66] as well as in SSCs of ovine testis [67]. SALL4 and PLZF interaction in SSCs provides a balance between stem cell self-renewal and differentiation. SALL4 acts as an antagonist to the PLZF transcription factor by removing it from its cognate sites, such as c-kit, which plays an important role in the differentiation pathway [2,68]. Similarly to the expression of PLZF and SALL4, we noticed significant upregulation of CD14. A recent study has demonstrated the expression of this marker in porcine testes by undifferentiated spermatogonia co-expressing also PGP9.5. In addition, CD14 expression has also been confirmed in cultured porcine SSCs [69], which agrees with our findings for cultured rabbit germ cells. Since the intracellular markers such as PLZF or SALL4 are not suitable for the direct enrichment of viable SSCs from the heterogeneous mixture of testicular tissue cells, a novel surface marker like CD14 might be a valuable choice for MACS or FACS isolation of rabbit SSCs. Using this marker, porcine SSCs have been successfully isolated [69]. ALDH, another popular marker for collecting stem cells from different tissue sources, has also been utilized for the FACS enrichment of mouse [31] or dunnart [32] SSCs using the ALDEFLUOR kit. However, both studies reported that ALDH-positive cells collected directly from dunnart testes or from the culture of mouse SSCs did not show overexpression of other SSC markers or better SSC activity upon transplantation, respectively. In fact, SSCs lack ALDH activity in vivo, but high expression of ALDH enzymes can be found in Sertoli and Leydig cells [31,32]. On the other hand, cultured mouse spermatogonia interestingly upregulated ALDH activity [31], which agrees with our observation for ALDH expression in cultured rabbit SSCs. It has been reported that a lack of vitamin A in culture media may increase ALDH expression, while high GDNF concentrations may alter the phenotype of in vitro cultured SSCs [31]. Anyway, high expression of ALDH by both rabbit SSCs and feeder layer cells, as shown by flow cytometry, indicated that this marker is not suitable for direct collection of SSCs from rabbit testes. On the other hand, ALDH-negative cells may also be enriched as potential undifferentiated spermatogonia, as reported by Tarulli et al. [32]. In addition to ALDH, cultured rabbit SSCs displayed overexpression of SSEA-4. Using this specific marker, a subpopulation of undifferentiated spermatogonia from human and porcine testes has been obtained with a typical SSC phenotype [70,71]. Thus, SSEA-4 might be another marker of choice for direct isolation of rabbit SSCs.

SSCs are primitive germ cells that, like other stem cells, are characterized by a large nucleus, less developed organelles or a lack of specific organelles typical for differentiated and mature cells, and a low level of protein synthesis. Cultured rabbit SSCs displayed a large spherical nucleus with dark heterochromatin spots under TEM, surrounded by round or oval mitochondria, vacuoles, and less abundant endoplasmic reticulum and lipid droplets. Similar ultrastructure has been found in SSCs of humans [72], horses [73], mice [74] and cattle [75], thus supporting the undifferentiated state of cultured rabbit germ cells.

In general, the ability of isolated SSCs to self-renew and differentiate into spermatozoa in vivo is usually verified by the spermatogonial transplantation method. This functional assay allows the transplantation of SSCs into the testes of an experimental animal model (e.g., germ-cell-depleted mouse) and evaluates the repopulating capacity of isolated SSCs by restoring spermatogenesis [76]. However, we were unable to verify the capacity of isolated rabbit SSCs, mainly due to the unavailability of the mentioned method in our laboratory. Thus, the lack of such a functional assay is the main limitation of this study and should be further considered for future studies, mainly in case of applying the rabbit SSCs for animal genetic resource conservation.

## 5. Conclusions

The presented culture system based on somatic testicular cells as a feeder layer provides not only physical support for cultured rabbit SSCs but potentially also ensures a sufficient level of molecular factors such as GDNF responsible for SSCs self-renewal, which together with their extrinsic supplementation provides optimal culture conditions for their proliferation, exhibiting typical SSC molecular markers. Moreover, a comprehensive phenotype of cultured rabbit germ cells has been explored, allowing the identification of several molecular markers, which might be used for the positive or negative enrichment of rabbit SSC subsets directly from testicular tissue (DBA^+^, CD14^+^, SSEA-4^+^, or ALDH^−^, respectively).

However, further studies are required to isolate rabbit SSCs by FACS or MACS according to suggested markers, to optimize this culture system for long-term proliferation and maintenance of rabbit SSCs, or to assess their repopulating capacity by functional assay in order to obtain enough pure SSCs from valuable rabbit breeds for their further cryopreservation and biodiversity protection.

## Figures and Tables

**Table 1:** Gene-specific primers and size of PCR products.

Gene	Product Size (bp)	Forward Primer	Reverse Primer	Reference
CD9	158	5′-CAACAAATTCCACGTCATCG-3′	5′-TTGAGGGGTACACCTTCCTG-3′	[37]
CD14	128	5′-TCTCTGTCCCCACAAGTTCC-3′	5′-GGCTGAGGTCTAGGTGATGG-3′	[38]
CD29	287	5′-AGAATGTCACCAACCGTAGCA-3′	5′-CACAAAGGAGCCAAACCCA-3′	[39]
CD49f	123	5′-AGGTACAGTGGTCGGTGAGC-3′	5′-TTCAAAGTTGCTGTGCCAAG-3′	[37]
CD90	293	5′-CTGCTGCTGCTCTCACTGTC-3′	5′-ACAGAAGCAGCTTTGGGAAA-3′	[39]
c-kit (CD117)	189	5′-CCGGTGGACTCCAAGTTCTA-3′	5′-GTAAACGTGGTGGGTGCTCT-3′	[37]
CDH1	168	5′-GAATCCTGGCTCTGCTCATC-3′	5′-GCTGGCTCAAGTCAAAGTCC-3′	[37]
SSEA-4	126	5′-CTGGGAGAATAACCGGTACG-3′	5′-GCTCAGTTGCCTCGGTAGAC-3′	[39]
ALDH	135	5′-CTGGGAAAAGCAACCTGAAG-3′	5′-AACACTGGCCCTGATGGTAG-3′	[28]
GFRα1	150	5′-CGAAATCCCCACTCATGTCT-3′	5′-TTTGTGGTTATGTGGCTGGA-3′	XM_051824180.1 ^1^
RET	93	5′-GGCGAAAGATGAAGATCTCG-3′	5′-TAACTGGAATCCGACCCTTG-3′	XM_051836896.1 ^1^
PLZF (ZBTB16)	125	5′-GCTGACCACTCCGCTTACTC-3′	5′-GGATCACTCACCGCAGGTAT-3′	XM_002708425.4 ^1^
PGP9.5 (UCHL1)	108	5′-CGGCCACCTCTATGAACTTG-3′	5′-GTGAATTCCCTGCACACCTT-3′	XM_002709402.3 ^1^
DDX4 (VASA)	93	5′-ATGAAGATGAAGGGGTGCAG-3′	5′-TGCTTGGGTAAGTGTCACCA-3′	XM_051853450.1 ^1^
DAZL	108	5′-CAGGCTGAGAAACTCCCTTG-3′	5′-AGCAGGGAGGATCAAACAGA-3′	XM_008266045.2 ^1^
SALL4	133	5′-GAGAGAGACCCTTCGTGTGC-3′	5′-GCCACTTTGTCCTGGAACTC-3′	XM_051832643.2 ^1^
NANOG	122	5′-GCCAGTCGTGGAGTAACCAT-3′	5′-CTGCATGGAGGACTGTAGCA-3′	[39]
OCT4	149	5′-GAGGAGTCCCAGGACATGAA-3′	5′-GTGGTTTGGCTGAACACCTT-3′	[39]
SOX2	152	5′-CAGCTCGCAGACCTACATGA-3′	5′-TGGAGTGGGAGGAAGAGGTA-3′	[39]
Vimentin	174	5′-GCGAGTACCAAGACCTGCTC-3′	5′-TTGAGTGGGTGTCAACCAGA-3′	[40]
SOX9	90	5′-GGCTCCGACACCGAGAATACAC-3′	5′-GAACTTGTCCTCTTCGCTCTCCTT-3′	[34]
B2M	118	5′-ATTCACGCCCAATGATAAGG-3′	5′-ATCCTCAGACCTCCATGCTG-3′	[39]

^1^ NCBI reference sequence.

## Data Availability

The original contributions presented in this study are included in the article. Further inquiries can be directed to the corresponding author.

## Abbreviations

The following abbreviations are used in this manuscript:

SSCs	Spermatogonial stem cells
FACS	Fluorescence-activated cell sorting
MACS	Magnetic-activated cell sorting
DMEM	Dulbecco’s Modified Eagle Medium
GDNF	Glial cell line-derived neurotrophic factor
GFRα-1	GDNF family receptor alpha-1
RET	Receptor tyrosine kinase
STO	SIM mouse embryo-derived thioguanine and ouabain resistant fibroblast cell line
PLZF	Promyelocytic leukemia zinc finger
PGP9.5	Protein gene product 9.5
CD90	Cluster of differentiation 90
Thy-1	Thymocyte differentiation antigen 1
DBA	Dolichos biflorus agglutinin
VASA/DDX4	DEAD-box polypeptide 4
NANOG	Transcription factor related to the pluripotency of stem cells
POU5F1/OCT4	POU domain, class 5
DAZL	Deleted in azoospermia-like protein
ITGA6	Integrin subunit alpha 6
CD49f	cluster of differentiation 49f
CDH1	Cadherin 1
ALDH	Aldehyde dehydrogenase
FGF2	Fibroblast growth factor 2
NZW	New Zealand White
PBS	Phosphate-Buffered Saline
MEM	Minimum Essential Medium
EDTA	Ethylenediaminetetraacetic acid
TEM	Transmission electron microscopy
